# An experimental comparison between primer and nucleotide labelling to produce RPA-amplicons used for multiplex detection of antibiotic resistance genes

**DOI:** 10.1038/s41598-023-42830-7

**Published:** 2023-09-21

**Authors:** Christian Warmt, Lisa-Marie Broweleit, Carolin Kornelia Fenzel, Jörg Henkel

**Affiliations:** 1 Fraunhofer Institute for Cell Therapy and Immunology - Bioanalytics and Bioprocesses (IZI-BB), 14476 Potsdam, Germany; 2 Institute for Biochemistry and Biology, University of Potsdam, 14476 Potsdam, Germany

**Keywords:** Chemical modification, DNA, RNA, Lab-on-a-chip, PCR-based techniques

## Abstract

Direct labelling of amplification products using isothermal amplification is currently done most frequently by incorporating previously labelled primer. Although this method is well proven and widely used, it is not a universal solution due to some weaknesses. Alternatively, labelled nucleotides could be used, whose application and functionality have been already partially demonstrated. It remains to be determined how this method performs in comparison to traditional labelling, in particular combined with isothermal amplification methods. In this work, we show a detailed analysis of the labelling efficiency under different conditions and compare the results with the traditional primer-labelling method in the context of RPA amplification. Impressively, our results showed that using Cy5-labelled dUTPs can achieve much more efficient labelling for fragments above 200 bp, while using them for smaller fragments does not bring any relevant disadvantages, but also no major benefit. Furthermore, this work successfully demonstrate for the first time a quadruplex microarray for the detection of resistance genes using RPA and direct labelling with Cy5-dUTP as a potential application scenario. The sensitivities achieved here extend to SNP discovery for the detection of the proper *bla*_KPC_ variant.

## Introduction

A variety of culture-based, biochemical and molecular biological methods for the detection of pathogens have been available for a long time^1–5^, in particular, the differentiation of these pathogens into their respective species and subspecies. On the level of molecular biology, the detection of specific nucleic acid sequences of various pathogens is commonly utilized. The majority of these methods have one thing in common: amplification of the relevant gene segments is required for sufficiently sensitive detection^6^. As demonstrated not at least by the detection of SARS-CoV-2 over the last three years, this is currently achieved mainly by polymerase chain reaction (PCR)^7^. However, in the context of point-of-care (PoC) or lab-on-chip (LoC) applications, but also in largescale laboratory automation projects, this method often comes up against technical and financial limits, since precise heating and cooling in cycles of seconds involve an enormous technical and energetic effort^8^.

In order to circumvent these and other disadvantages of PCR (e.g. amplification time and the mandatory trained staff), various alternative methods are available, which are commonly referred to as “isothermal amplification” methods^9–12^. By using a wide variety of complex molecular reaction mechanisms with specific enzymes and strand displacing polymerases as well as special designed primer sets, all of these methods allow DNA to be amplified at a constant and low temperature without the energetically and technically expensive thermal cycling of the PCR process^13^.

However, this technical advantage is accompanied by various procedural difficulties, which in turn affect the detection and evaluation of the amplification products. A known problem of recombinase polymerase amplification (RPA) is the occurrence of false-positive amplification products^14^. Loop-mediated isothermal amplification (LAMP), on the other hand, results in products of varying lengths with repeating base sequences (concatemers)^9,15^. Due to these circumstances, a simple evaluation of the results by agarose-gel-electrophoresis is often not possible. This is further supported by the fact that gel analysis is not optimally suited for LoC or PoC applications. Therefore, alternative methods such as lateral flow analysis (LFA)^16,17^ or microarray technologies^18–20^ are often used.

However, these alternative methods require amplicon labelling with different molecules in the overall process. During microarray technology, a fluorescent dye is often incorporated into the target DNA for subsequent detection^21^. In addition to labelling with a fluorophore, LFA requires further labelling with biotin^22^.

**Figure 1:**
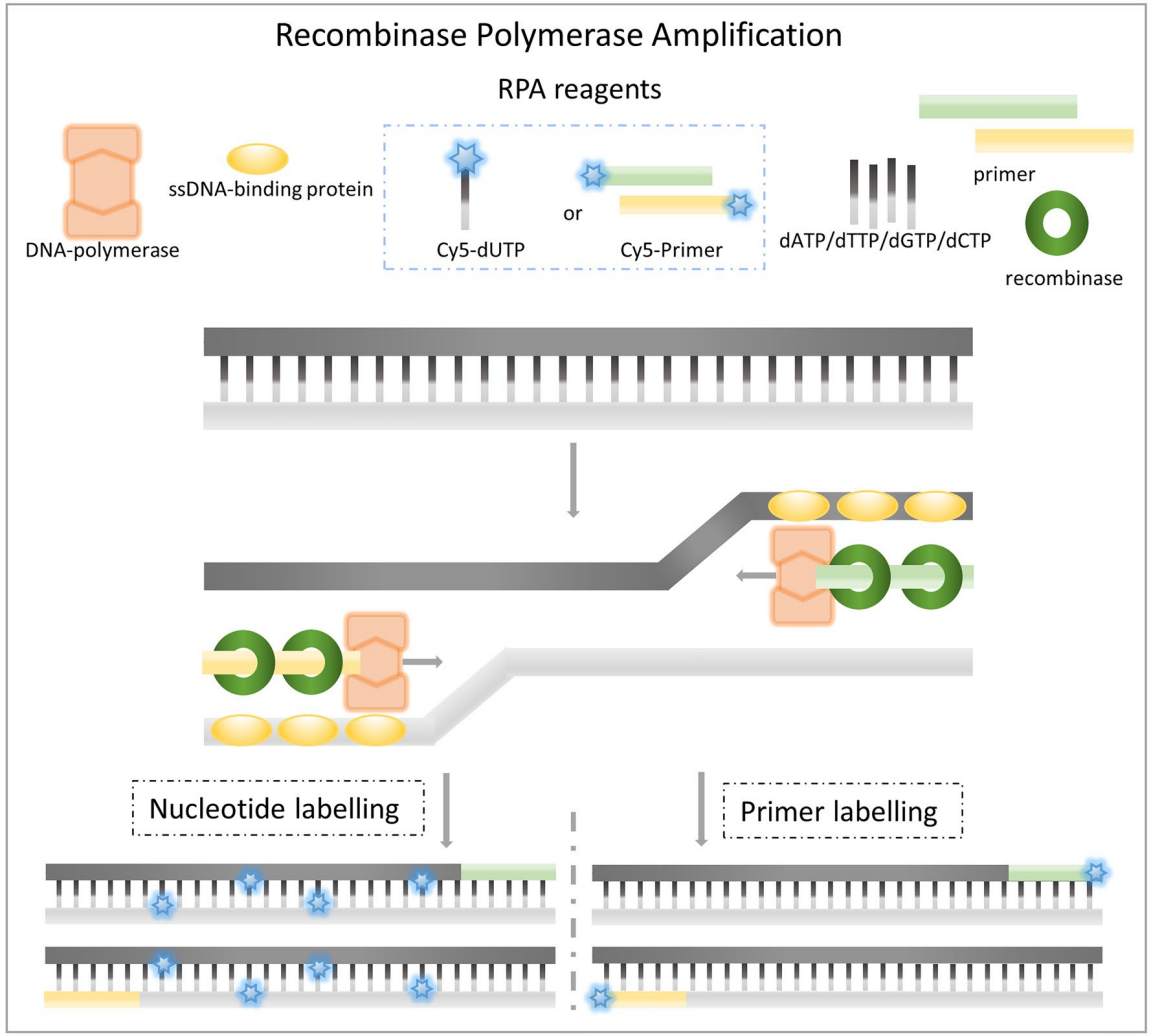
DNA amplicon labelling during RPA. Illustrated are the two most fundamental options for labelling amplicons directly during RPA. In addition to the DNA-polymerase, recombinase and ssDNA binding proteins commonly used for RPA, as well as the unlabelled dNTPs and primer, dUTPs or primer labelled with fluorophores are also required (blue dashed box). After being amplified, there are either (several) nucleotide labels (left side) or one primer label per ssDNA in the RPA product.

The aforementioned labelling usually takes place during amplification of the genetic material. On the one hand, labelling can be carried out via labelled primer^23,24^ and, on the other hand, via labelled nucleotides^25–28^ (Fig. 1). The former seems to be considered a kind of dogma in the scientific world and can be found in far more than 90% of amplification-based publications. Even though this method is used frequently, its use is comparatively inflexible and the fluorescence intensity is limited to one fluorophore per single-stranded DNA (ssDNA) amplicon. Thus, it is not always the perfect instrument of choice, especially for procedures that require maximum sensitivity. Not only because of this, more sensitive and expensive optical devices may therefore be required for subsequent detection, or alternatives must be found to increase the overall sensitivity of the assay.

The use of nucleotide-based labelling methods can significantly increase the labelling efficiency and thus the sensitivity of an assay^18^.

The principle as well as the effect of labelling efficiency when using different labels by means of deoxynucleoside triphosphates (dNTPs) for PCR and LAMP^18,22^ could already be shown in different studies.

Only a few studies have shown the incorporation of modifications using labelled nucleotides during RPA. In these, the incorporation of tyrosine and tryptophan^29^ but also of ferrocene-labelled nucleoside triphosphates^30^ for electrochemistry has already been successfully demonstrated.

Biotin modifications can also be incorporated into the RPA products to enable subsequent coupling with enzymes or magnetic beads via streptavidin and final optical or electrochemical detection^31,32^.

However, the molecules are predominantly small molecules that may be less inhibitory than large fluorophores during amplification, as we were able to show in a LAMP based study^18^. Furthermore, in many cases (e.g. after biotin coupling) a further coupling step is necessary before the actual detection. Neither of the methods to date show direct, nucleotide-based fluorescent labelling of the amplicons for immediate detection on the microarray during RPA, nor is their use in multiplex analyses being investigated.

In a proof of concept study, we have been the first to successfully demonstrate the use of fluorescent dNTP-based labelling within the RPA procedure followed by microarray technology^33^.

Despite this, our previous study on labelling of RPA products was only a basic proof-of-concept that fluorescent nucleotide labelling is also possible in RPA. It is unclear whether the data from the LAMP-based studies^18^ can also be applied to RPA. In addition, this study aims to clarify whether an extension of the application to a multiplex RPA using labelled nucleotides to detect genes not previously detected with this method is also possible and, above all, useful. A corresponding study on this has not yet been found in the relevant literature and therefore represents a novelty.

In the present study, the usage of Cy5-labelled nucleotides in RPA will be further investigated and compared to commonly used primer labelling. Both, fragment length dependencies and economic aspects are considered.

In particular, the consideration of economic aspects should play a major part in this. They should make it easier for the scientific community to decide which method is the most appropriate for them without having to invest time, money and other resources in their own basic experiments. Therefore, these aspects should be presented and discussed here to a relevant extent in order to provide the research community with a simple and quick overview and comparison of the two possible labelling methods. This comparison is missing in the literature so far, both in the field of PCR and RPA, as well as in other isothermal methods.

The amplification and detection of different genes from different organisms at the same time in a single reaction (multiplex analysis) is always a challenge. Reagents, buffers and concentrations often have to be adjusted. The system that works for a simple analysis may need to be optimised. The use of labelled primers in the multiplex procedure is described in both PCR^34^ and RPA^21^. The use of fluorescent-labelled nucleotides for this purpose has not yet been documented in the literature for RPA until today. It is therefore unclear what effect this may have on labelling. Filling this knowledge gap is a further aim of the present work.

For the first time, we demonstrate in this work a quadruplex RPA with downstream microarray detection and integrated single nucleotide polymorphism (SNP) analysis in which the analytes were not labelled via primer but via dNTPs during amplification.

## Results

### Comparison of Cy5 incorporation rates: nucleotides vs. primer

For a detailed analysis of the incorporation rates and the validation of the conditions under which the use of labelled nucleotides or labelled primer shows promising results, different RPA reactions with varying conditions were performed. Using a Cy5-dUTP concentration series (2–80 µM), the RPA was performed with fragments of different lengths and resistances (here exemplified on *bla*_CTX-M15_ with 141 bp fragment and *bla*_KPC_ with 809 bp fragment). An initial optical analysis was carried out by visual observation of the amplicons on an agarose gel.

After RPA and subsequent purification using magnetic beads, the concentration of the amplicons was also determined. The concentration remained constant over the entire range of Cy5-dUTPs used, with no tendency to increase or decrease, averaging 82 ng/µl (CTX-M15) and 155 ng/µl (KPC) for 60 min RPA and 25 ng/µl (CTX-M15) and 20 ng/µl (KPC) for 40 min RPA. The concentrations determined using the labelled primer were only 40–65% of the samples amplified with Cy5-dUTP after purification of the amplicon in all test series.

**Figure 2:**
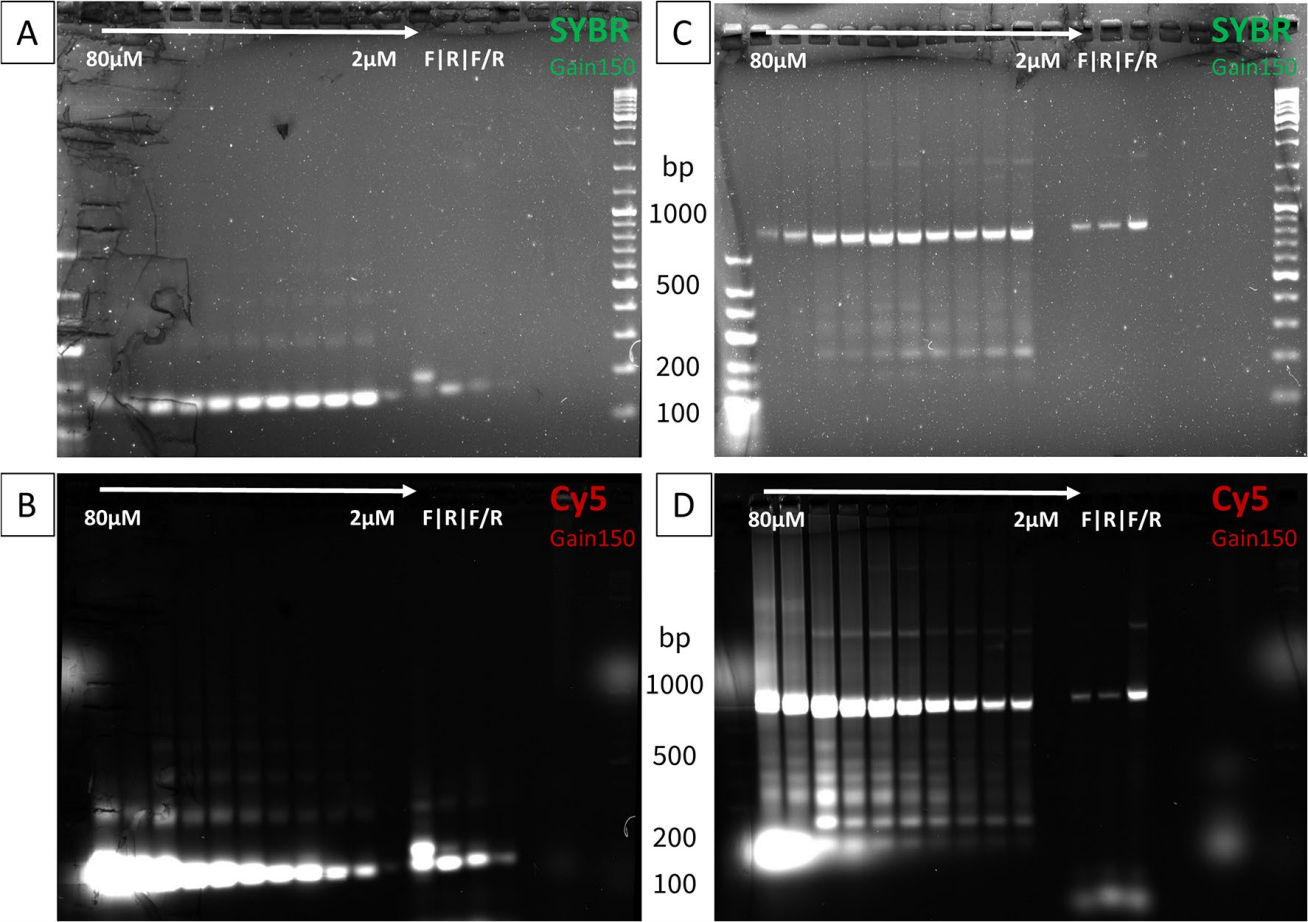
Detection of fluorophore incorporation into RPA amplicons by gel electrophoresis. Shown is the incorporation of Cy5-dUTP with using different concentrations during RPA (2 µM to 80 µM) and by replacing the unlabelled with labelled forward primer (F), reverse primer (R) and both primer (F/R) simultaneously. A=*bla*_CTX-M15_ amplicon and C=*bla*_KPC_ amplicon on common gel images using the intercalator SYBR-green are shown. B=*bla*_CTX-M15_ amplicon and D=*bla*_KPC_ amplicon: all RPA products in which the dye Cy5 has been incorporated are visible on the same gels.

For the examination of the electrophoretic gels, a microarray scanner with a modified mounting was used to hold the gels. This allowed to be viewed both under normal conditions (viewing all DNAs via an intercalator) and under excitation with a wavelength of 633 nm (Fig. 2). The latter was used to visualize only those DNA fragments that actually incorporated Cy5. The images for both the CTX-M15 fragment and the KPC fragment showed bands at a height of about 150 bp and 800 bp, respectively, spanning the entire Cy5-dUTP concentration range and using the labelled primer. These were detectable at both 532 nm (SYBR green) and 633 nm excitation. For both the CTX-M15 fragment and the KPC fragment, there were additional, predominantly weaker bands detected above and below the 150 bp and 800 bp.

While the observation of the gels under SYBR green conditions showed uniform band intensities for the Cy5-dUTP concentration series and minimally weaker intensities for the primer samples, a different occurrence was observed in some cases under Cy5 conditions. In particular, for the smaller CTX-M15 fragment, it could be seen that despite the low intensities in the SYBR green image, the bands in the Cy5 image of the primer-labelled fragments provided stronger signals than the lower concentration range of the Cy5-dUTP series. In contrast, for the KPC fragment, the primer-labelled bands in both the SYBR-green image and the Cy5 image are weaker or at least equally intensive as the lower Cy5-dUTP concentrations.

For both the CTX-M15 fragment and the KPC fragment, it is obvious that the Cy5 intensity in the amplicons increases with increasing Cy5-dUTP amounts in the RPA assay.

In addition to the KPC bands at the 800 bp level, a Cy5 fluorescent blob at the 150 bp level can be seen in the gel, which strongly increases in intensity towards the 80 µM Cy5-dUTP sample and is at the same level as the 40–80 µM Cy5-dUTP signals in the CTX-M15 image. In comparison, no DNA band is visible in the SYBR green image.

**Figure 3:**
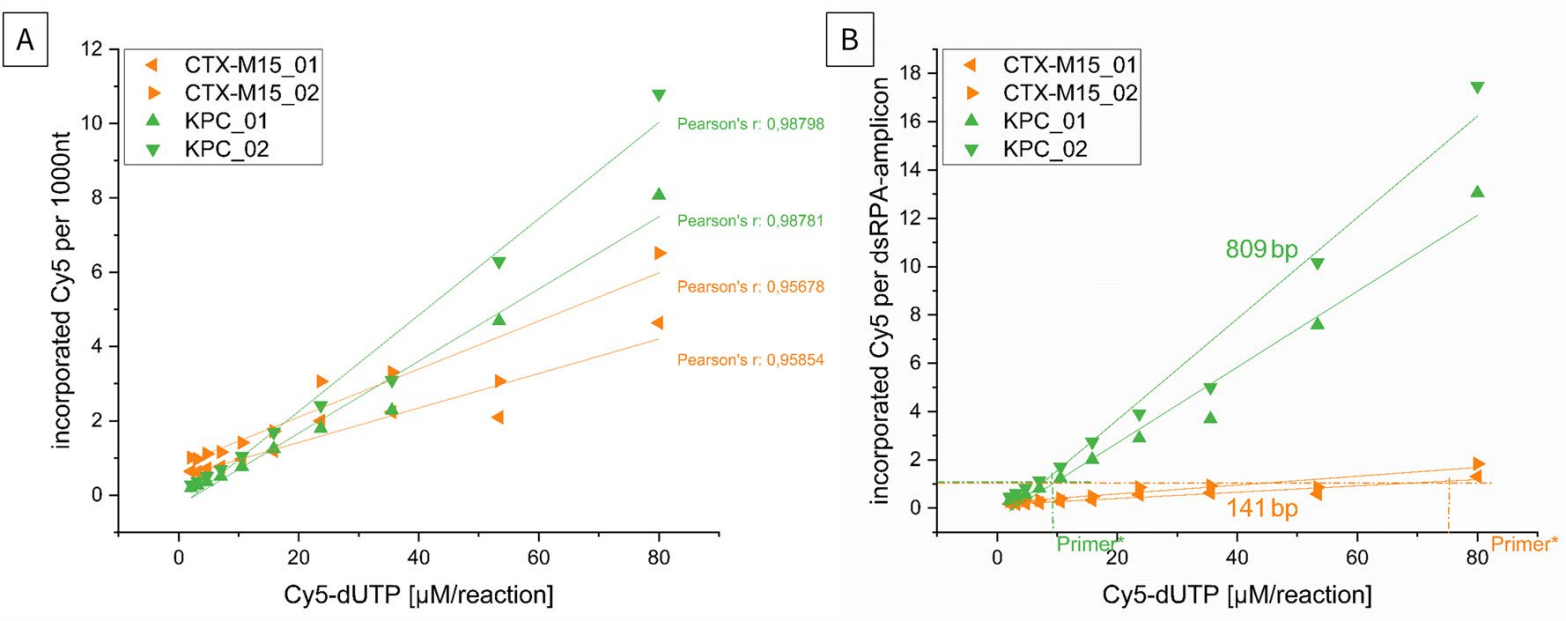
Comparison of Cy5 incorporation rates as a function of RPA fragment length. The Cy5 incorporation rates of the two fragments of varying lengths for the detection of *bla*_CTX-M15_ (141 bp) and *bla*_KPC_ (809 bp) are shown. A: indicates the incorporation rates per 1000 nucleotides used for the formation of DNA amplicons during RPA as a function of the Cy5-dUTP concentration applied. B: shows the absolute incorporation rate of a double-stranded RPA amplicon relative to the respective fragment length and Cy5-dUTP concentration used per 25µl RPA reaction. The dashed lines indicate the amounts of Cy5-dUTP required to theoretically achieve the same incorporation rate as when using one labelled primer.

Although the gel electrophoresis provides an informative visual overview of Cy5 incorporation under varying conditions, the incorporation rate (Fig. 3) is also of interest for a detailed analysis. In order to calculate the corresponding incorporation rates, the previously purified RPA samples were measured by fluorescence spectroscopy. To ensure that the RPA would amplify to the endpoint, a comparatively high template concentration of 1 ng PCR product per RPA and an amplification time of 60 min were used. It was found that the estimated incorporation rates per 1000 nucleotides differed depending on the fragment length and with increasing Cy5-dUTP concentration per RPA (Fig. 3A). Thus, it can be seen from the calculations that at both fragment lengths and a Cy5-dUTP concentration up to 30 µM/RPA, an incorporation of 1.5–3 fluorophores per 1000 nt can be achieved. At a concentration of 80 µM Cy5-dUTP, the concentrations vary between 4 and 5 fluorophores per 1000 nt for the small fragment and 8–11 fluorophores for the large fragment. In all experimental series, linearity between the incorporation rate and the nucleotide concentration used could be determined with a Pearson correlation of r = 0.957 to r = 0.988.

Considering the amount of fluorophores incorporated during RPA relative to the actual fragment length of the double-stranded amplicons (dsDNA), an even greater difference in the incorporation rates between the short and the long fragment stands out (Fig. 3B). Thus, it can be seen that the short CTX-M15 fragment at 20 µM, 40 µM and 80 µM 0.5, 1 and up to 2 fluorophores could be integrated. For the 809 bp fragment of KPC, the amount of fluorophore incorporated was 4, 8 and up to 18 molecules at the same Cy5-dUTP concentrations.

This leads to the additional observation that for the small 141 bp fragment, a concentration of 70–75 µM Cy5-dUTP is required to achieve the same labelling efficiency (1 molecule per dsDNA) compared to using one labelled forward or reverse primer each. With the 809 bp amplicon, only 15 µM is required. All concentrations above this exceed the maximum labelling efficiency possible with primer labelling.

### Labelling of RPA amplicons for microarray detection

**Figure 4:**
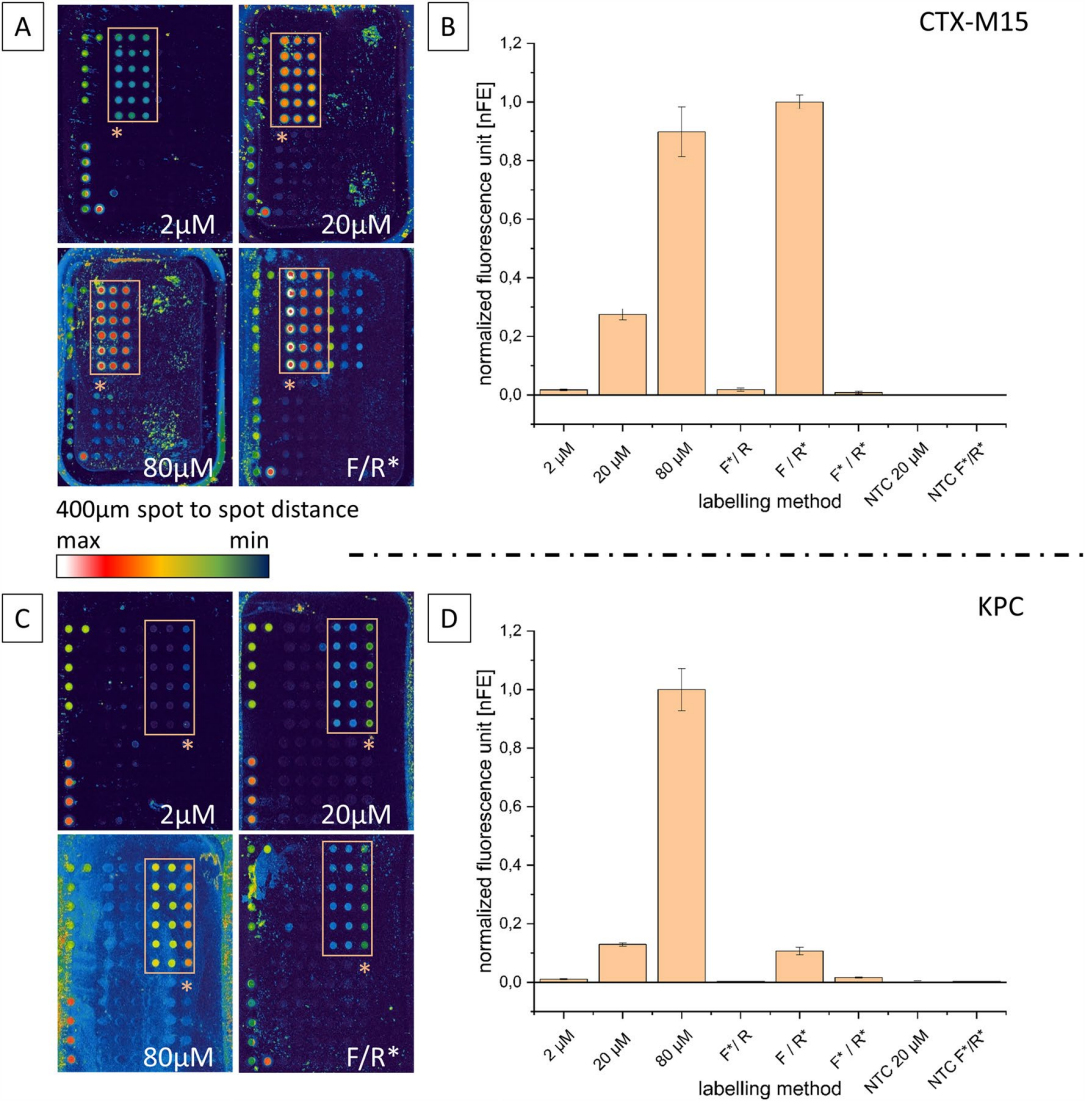
Comparison of nucleotide and primer labelling with varying fragment size. Shown is a comparison of the fluorescence intensities of two fragments of different sizes (CTX-M15 fragment = 141 bp; KPC fragment = 809 bp) after RPA and detection via microarray. (**A**)/(**C**): Microarray false colour representation for the detection of blaCTX-M15 and blaKPC after labelling with 2 µM, 20 µM and 80 µM Cy5-dUTP and labelled reverse primer (F/R*); orange frame marks fragment-specific probes. (**B**)/(**D**): Graphic representation of the microarrays shown on the left (probes marked with orange asterisk) supplemented with the negative controls (NTC) and the remaining primer labelling methods (F* forward primer; F*/R* both primer; scan parameter: gain 500, laser power 50, filter standard red).

For a rapid and easy analysis by means of Nucleic Acid Amplification Technology (NAAT), which may even have to take place on site and not in a fully equipped laboratory, measurement and evaluation by using fluorescence spectroscopy is rather impractical. Therefore, the findings on the incorporation of the Cy5 molecules should be performed on a more realistic example, the microarray analysis after an RPA. For this purpose, 1.0–1.5 ng templates were again amplified for 40 min at 37 °C and then analysed via microarray without further follow-up treatment. For this purpose, each array contained three specific probes for the CTX-M15 and KPC amplicon (Fig. 4; microarray false colour representation, orange frame).

The microarray representation of the CTX-M15 fragment, which is only 141 bp in size, shows that a Cy5-dUTP amount of 2 µM is sufficient to detect the amplicons. However, the detectable signal intensities are comparatively low, recognisable by the green-bluish spot colours in the false colour representation. The intensities reach only about 2–3% of the results achievable with primer labelling (Fig. 4; bar chart CTX-M15).

Using 20 µM Cy5-dUTP, this can be increased to 25–30%, achieving the same signal intensities as using labelled reverse primer for this short fragment at about 80 µM. Both the negative controls for Cy5-dUTP and primer labelling and the sample with the labelled forward primer showed weak or even no fluorescence. Using the two labelled primer, only a weak signal intensity could be detected in repeated experiments.

Slightly different results revealed when looking at the KPC microarray. Once more, the 2 µM Cy5-dUTP RPA approaches show the weakest signals, followed by the 20 µM and 80 µM approaches. However, the signal intensities using the reverse primer are about the same level when using the 20 µM approach. Thus, the intensities of the primer labelling are about 10–12% compared to the use of 80 µM labelled nucleotides.

In case of the detection of the KPC fragment, also no signals are detectable in the negative controls and the labelling with the labelled forward primer. The use of both primer again gives significantly weaker signals than the use of the single reverse primer.

For the bar chart in Fig. 4 (section KPC), the signals of the right outer probe (KPC_S_C) were used. Compared to the other two probes, this one had a higher intensity, which can be seen in the false colour representation by the more intense green to orange colouring. The probe used here is a SNP probe designed to detect the KPC-2 variant.

### Performance of nucleotide labelling in multiplex analysis

For the detection of various target genes, in particular for the determination of antibiotic resistance, it is helpful to perform a multiplex analysis, both in terms of technical effort and cost. Therefore, the Cy5-dUTP RPA microarray assay developed here was investigated for its multiplex capability to detect various resistance genes. For this purpose, the analyses were performed on purified genomic material (1 ng template; 20 µM Cy5-dUTP/RPA reaction) rather than on pure PCR products. Four different resistance genes from four different organisms (*bla*_CTX-M15_ [*E. coli*; 735/14–1], *bla*_NDM_ [*E. coli*; 2/10], *bla*_VIM_ [*P. aeruginosa*; 359/11] and *bla*_KPC_ [*E. coli*; 17/11]) were detected.

In a preliminary analysis, any cross-reactions of the different RPA primer and microarray probes should be excluded. For this purpose, two of the resistance genes were amplified individually and simultaneously using the respective RPA primer in combination.

**Figure 5:**
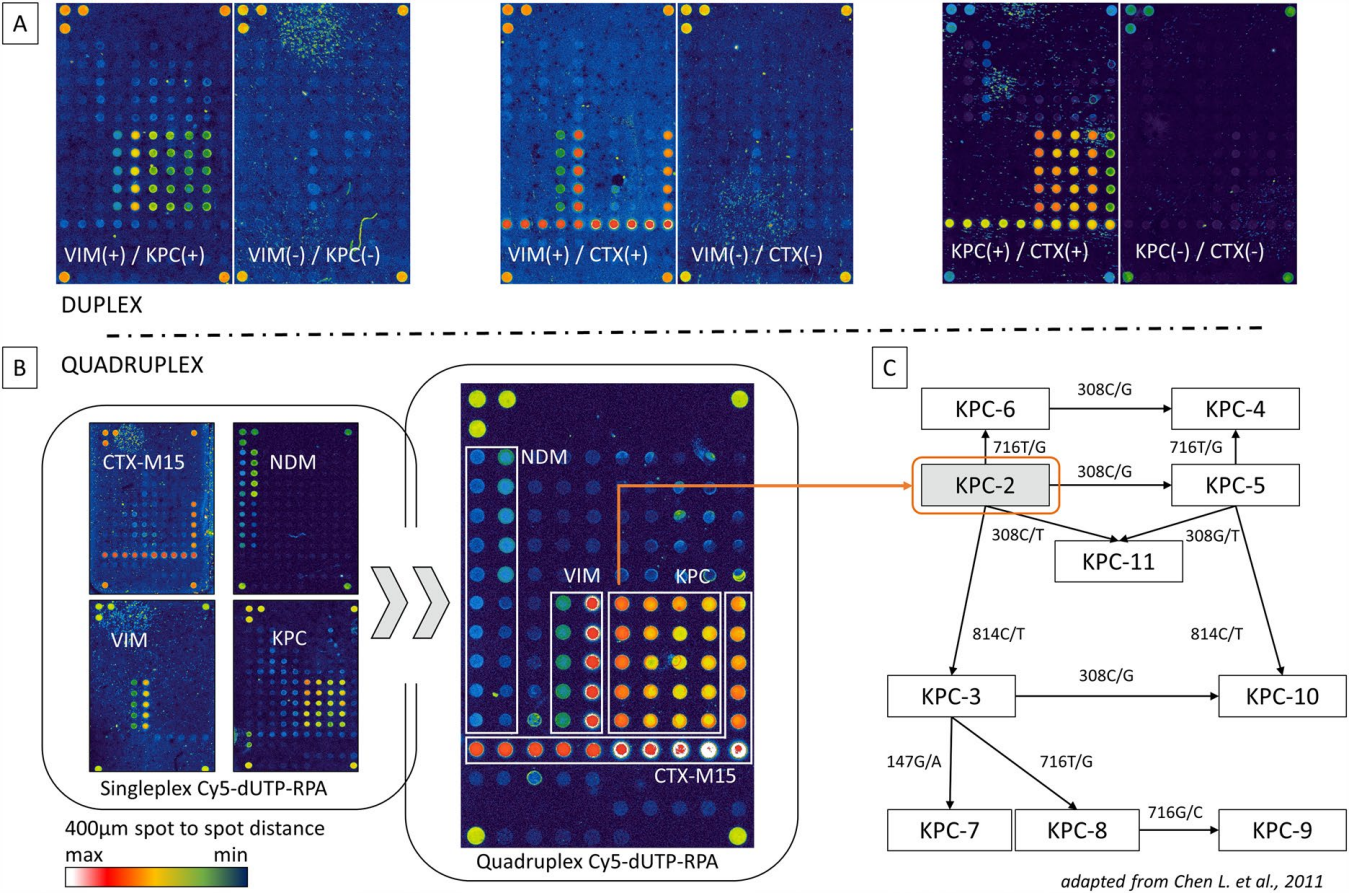
Duplex and Multiplex detection of four resistance genes by Cy5-dUTP labelling. Depicted are multiplex RPA assays combined with microarray detection for the simultaneous detection of up to four different resistance genes from four different organisms (*bla*_CTX-M15_ [*E. coli*; 735/14-1], *bla*_NDM_ [*E. coli*; 2/10], *bla*_VIM_ [*P. aeruginosa*; 359/11] and *bla*_KPC_ [*E. coli*; 17/11]). (**A**): Duplex experiments to validate possible cross-reactions are shown; (+) indicates which primer combinations and associated templates were used during the RPA; (−) indicates the negative controls using the respective primer but without templates. The positions of the respective probes are shown in the singleplex figure (**B**). For each microarray probe, a fivefold determination (five spots in a row or in a line) was performed. (**B**): Results of the quadruplex approach. The positions marked in the quadruplex for the probes of the respective amplicons are identical in all figures. Both in the single and multiplex approach, the *bla*_KPC-2_ variant can be detected via the high signal probe (left outer probe in the KPC block; orange arrow guides to KPC-2). Microarray: false colour representation. (**C**): Schematic representation of KPC variants based on varying SNPs according to Chen L. et al., 2011.

It was found that in case of a simultaneous detection (Fig. 5; top panel; duplex) for KPC and VIM amplicons, both probes for the *bla*_VIM_ gene and the four probes for the *bla*_KPC_ gene gave a signal in the false colour representation, though of different strength. The positions of the respective probes can be seen in the singleplex figure below (Fig. 5; lower panel; singleplex). The probes for the KPC analysis almost entirely show a weak to medium signal intensity (green coloration), while one of the two VIM probes shows a medium strong (yellow-orange) and the second one a very weak (light blue) intensity. A similar picture is shown by the images of the simultaneous analysis of the VIM and CTX-M15 fragment. In general, the VIM signals appear stronger here (indicated by the more intense red coloration), but again the left probe is much less fluorescent. The CTX-M15 signal strengths of the different probes are on the same level. Only slight signal differences can be seen here.

The combined detection of *bla*_KPC_ and *bla*_CTX-M15_ also appears similarly. In none of the shown cases false positives were observed, neither in the individual analyses using both RPA primer (not shown) nor in the no-template controls (NTC).

In a following and final analysis it should be determined whether a quadruplex RPA with subsequent microarray detection for the simultaneous detection of four different target sequences and prior Cy5-dUTP labelling can be performed with success. For this purpose, in addition to the residue genes *bla*_KPC_, *bla*_CTX-M15_ and *bla*_VIM_, the additional gene *bla*_NDM_ should also be detected. The corresponding primer for all four genes were used simultaneously in the RPA and the amount of Cy5-dTUP used was also 20 µM using 1 ng genomic DNA from each of the four different bacterial strains. The results are likewise shown in Fig. 5 (Quadruplex Cy5-dUTP-RPA) as a false colour representation of the microarray.

As already shown in the duplex assays, different fluorescence intensities can be detected in the quadruplex microarray at the corresponding positions where the specific probes for the four genes are located. This is not only true for the already shown 2 probes for the VIM fragment, the 3 probes of the CTX-M15 fragment and the 4 probes of the KPC fragment but also for the 4 probes of the newly introduced NDM fragment. These NDM probes show the weakest fluorescence signals, followed by the left VIM probe and the KPC probes. The right VIM probe and the two lower CTX-M15 probes have the strongest fluorescence signals and are within the scanner's saturated detection range at the given scanner settings (indicated by the white-coloured spots). Thus, for the multiplex assay, a signal intensity is present over the entire detectable range of the microarray scanner used.

Furthermore, it can be seen that of the four KPC probes used, the probe KPC_S_C (on the very left) delivers stronger signals than the remaining three. Thus, also in the case of quadruplex detection, this probe points to the mutation 308C/T in the *bla*_KPC_ gene for the resulting KPC-2 variant.

## Discussion

In the field of nucleic acid amplification technologies (NAATs), isothermal methods, which can avoid cyclic annealing of the amplification reaction as in PCR by means of sophisticated molecular biology techniques, are becoming increasingly popular. Besides loop-mediated isothermal amplification (LAMP) and rolling circle amplification (RCA), recombinase polymerase amplification (RPA) remains the third most widely published and used isothermal amplification method^13^.

In combination with various detection methods such as microarray technology or lateral flow analysis (LFA), this method is perfectly qualified for on-site diagnostics and point-of-care applications, not at least because of its amplification temperature of only 37 °C^35^.

Nevertheless, in order to detect the RPA amplicons after the reaction, they first must be labelled. Usually, this is realized by using labelled primer.

However, another way is simply to replace the labelled primer with labelled nucleotides.

We have already shown in previous works that the use of labelled nucleotides can certainly have advantages over primer labelling when isothermal amplification methods are used^18,22^. Also, the use of Cy5-labelled dNTPs to highlight amplicons during RPA has already been successfully demonstrated as a proof of concept^33^.

Despite the possibility of using labelled dNTPs, not only for fluorescent labelling but for general labelling with a variety of different markers, up to now the primer variant is most commonly used and certainly represents a sufficient method for the vast majority of tasks. Therefore, this work does not claim to fundamentally question this method, but is simply intended to show an alternative and to demonstrate by means of concrete examples when a change to nucleotide labelling should be considered. Although a few papers have been published on labelling amplicons via dNTPs, there does not appear to be any comparative work on this topic to date regarding PCR and certainly not in the area of isothermal amplification.

The labelling of amplicons via nucleotides certainly brings one or the other advantage. For example, a faster and more flexible adaptation of the assay to new primer or other labels is possible without the need to relabel each new primer. This can save time and cost, especially in the case of complex and time-consuming procedures.

However, the most evident advantage of the labelling with nucleotides is the possibility of multiple labelling. While only one modification per ssDNA or two per dsDNA can be inserted into the amplicons when using primer, it should be possible to vary the labelling rate when using different dNTP concentrations. This fact could also be shown previously using LAMP products^18^. Thus, in this work, different concentrations of 2–80 µM Cy5-dUTPs, in addition to the unlabelled nucleotides, were added to the RPA set-up. It was noticed throughout all experiments that an increase in the dUTP concentration was always followed by an increase in the fluorescence signals. Already when looking at the amplicons on the gel, this effect was seen, both for CTX-M15 and KPC. At approximately constant concentrations of RPA product, a strong increase in the signals towards the variants with a high amount of Cy5-dUTP in the RPA assay could be seen when looking at the Cy5 scans. However, it is also interesting to note at this point that in the case of a small (smaller than 150 bp) fragment, the minimum concentration of 20–40 µM Cy5-dUTP was required in the RPA to achieve the same signal intensities on the gel as with the single or double primer labels (Cy5-forward, Cy5-reverse or both). This effect was clearly visible despite the apparent smaller amount of DNA template of the primer-labelled amplicons of CTX-M15.

Using a larger (greater than 800 bp) fragment, a different result was obtained. Here, even the smallest Cy5-dUTP concentration of 2 µM could produce stronger fluorescent bands. The analyses of the gel images therefore gave a first insight into how much the signal intensity depends on the length of the amplified fragment.

In fact, this circumstance was also apparent in the calculation of the incorporation rates. Based on these, it becomes clear that for the short fragment with a length of 141 bp, an amount of 70–80 µM Cy5-dUTP would be necessary in the RPA preparation to obtain the same incorporation rates as when using labelled primer. In contrast, for a fragment approximately 800 bp long, only 15–20 µM is required.

These findings were observed both by fluorescence spectroscopy, that was the basis for the calculation of the incorporation rates, and in the later microarray experiments (Fig. 4). Again, a significantly higher concentration of Cy5-dUTP was required for the small RPA fragment than for the long fragment.

In contrast to expectations formed by the gel image, using forward and reverse primer at the same time did not increase the fluorescence. Also, an approximately equal intensity on the microarray as when using only one labelled primer (only the strand complementary to the probe generates a signal) would have been expected here. Here it seems that the use of two primer has a stronger negative influence on the amplification. The exact reason for this could not be completely clarified so far and will be the subject of further investigations.

Thus, for the CTX-M15 fragment using 80 µM labelled nucleotides, a maximum labelling of 2 fluorophores per dsDNA and for the KPC, an incorporation of up to 18 fluorophores is possible. A calculation of the incorporated Cy5 molecules to 1000 nt should result in the same values independent of the fragment length. However, it turned out that this was only true for the range around 15–20 µM Cy5-dUTP. In the higher concentration range, the longer KPC fragment showed an incorporation rate somewhat twice as high as that of the short CTX-M15 fragment. The reason for this is probably the by-products, which can often occur in RPA. While these are of minor importance when using a suitable detection method (e.g. specific probes for LFA, microarray or qRPA), they still hamper the calculation of the true incorporation rate here. As a result, incorporation rates for products with by-products larger than the target, such as occurs with CTX-M15 amplification, tend to show lower incorporation rates than actually exist. In the reverse case, as in the KPC amplification example, higher incorporation rates are tendentially calculated. Therefore, the calculations presented here should only be seen as approximate values. A comparison of the two methods, primer vs. nucleotide labelling, is nevertheless possible and valuable in this context, since mainly the truly measurable fluorescence signals will be evaluated and compared.

When considering the two different labelling methods during RPA, not only the incorporation rate but also the influence on the amplification process is of crucial importance for later decisions. In the present experimental setup, we could not detect any significant influence of the two methods on the amplification rate or yield of the RPA products. After purification with magnetic beads, small variations could be measured, but they had no distinct tendency and were probably more due to the variations in the purification method. Furthermore, RPA was investigated at both 40 min and 60 min. No differences were observed when using different Cy5-dUTP concentrations. Even with 80 µM Cy5-dUTP, the RPA worked sufficiently well. When comparing primer labelling and nucleotide labelling, there was a slight tendency for slightly reduced amounts of product to occur when using the primer. However, these obtained values are not significant enough to conclude that dUTP labelling generates better product yields.

The method of fluorescence labelling used here is of course only one possibility to label the RPA products depending on the detection method or subsequent processing steps. Therefore, this is only an example for a multitude of other possibilities, such as labelling with biotin, dioxigenin or also NH_2_ to be able to couple the amplicons later to or with something different.

Depending on the label used, the cost of the total assay may be a relevant decision criterion. While the use of biotin-dUTP or NH_2_-dUTP with about 0.6 € and 0.03 € respectively have only a small or no influence on assay costs (when using 20 µM per 25 µl RPA), the situation is different for a fluorescence label. In addition to the RPA costs of about 2 € per assay (liquid basic kit), which are not inexpensive, a further 3.5 € are required if a 20 µM Cy5-dUTP labelling is to be performed. The use of labelled primer is much less expensive with about 0.1 € extra cost per preparation. In the end, the user has to decide whether the higher signal intensity is worth the higher costs.

An option to minimize the cost of nucleotide-based labelling would be a multiplex assay. In fact, using primer labelling, the price per RPA reaction increases with each new primer combination because each additional target requires its own labelled primer. Compared to nucleotide labelling, the assay price decreases with each new target, since the same nucleotides can be used for all amplification reactions. With the additional costs of about 0.1 € for simple primer labelling and 3.5 € for nucleotide labelling, this means that already with a quadruple multiplex the additional costs per total assay rise to 0.4 € or respectively fall to 0.85 €. With a 6-fold multiplex, both variants would even have levelled off to 0.6 €.

Whereas multiplex analyses by RPA amplification followed by microarray using primer labelling method has already been shown in the literature^21^, a quadruplex RPA with subsequent microarray detection was performed in this study to investigate whether nucleotide labelling can be used for this purpose.

It was demonstrated that the four different resistance genes from four different organisms could be successfully detected after simultaneous amplification via microarray. From previous studies and based on the results and cost estimates shown so far, a concentration of 20 µM Cy5-dUTP was used here for the approach.

Based on prior results, it would have been expected that the CTX-M15 fragment would show lower signals in this case, compared to the KPC fragment, as the Cy5 incorporation rates are much lower for this fragment. Nevertheless, CTX-M15 shows by far the best signals, even though it was the smallest fragment. This example demonstrates that, among other factors (e.g. fragment length, quality of genomic DNA, frequency of resistance genes per genomic DNA), the amplification efficiency per target and the specific probes on the array are just as relevant as the choice of labelling method.

Furthermore, in the quadruplex approach, as in the singleplex assay before, nucleotide labelling can be used to detect not only the *bla*_KPC_ gene as such but also the correct *bla*_KPC-2_ variant using SNP probes.

For both the microarray-based single detections and the multiplex analyses, there appear to be sporadic false-positive signals on the array. These are quite clearly visible to the human eye in the false colour display for better visualisation, but are usually not significantly more intense than the background noise or the signal intensities of the negative controls when viewing the raw data of the fluorescence signals. Nevertheless, an optimisation of the array, possibly also of the upstream RPA, is certainly conceivable and useful here. In this case, optimisation can be achieved above all with regard to the probes used or the washing protocol of the microarray. Optimisation of the sequences to be amplified during the RPA is also certainly conceivable in this context. This could possibly also minimise the previously mentioned by-products of RPA amplification. This optimisation work is the focus of subsequent studies and was not further investigated in this work, as the focus was on overall feasibility and the comparison of the different methods and their cost-effectiveness.

In conclusion, the use of nucleotides for labelling RPA amplicons directly during the amplification is a promising alternative to the common labelling via primer. However, the decision which of the two methods is the better one for the respective application must be made by each user himself, considering many factors. In addition to the fragment length and the type of detection method (e.g. LFA, fluorescence spectroscopy, fluorescence gel or microarray), the type of labelling molecules (e.g. fluorophore, biotin, functional groups) as well as a cost–benefit analysis (labelling costs vs. signal intensity) plays a significant role. Also, the fact whether and how receptive a multiplex analysis should be performed has to be included in the consideration.

Regarding the amplification efficiency and the speed of the RPA reaction, neither the use of one labelled primer nor the labelled nucleotides seems to have an advantage over the other, although there are indications of a slightly increased product yield, especially in the lower Cy5-dUTP. However, this issue may need to be investigated in more detail. The use of two labelled primer tended to show a decrease in signal. In this case, we would recommend the use of nucleotides instead of two primers to increase signal integrity.

As expected, both nucleotide and primer labelling have their advantages and disadvantages. However, from our point of view, it should be deviated more often from the current doctrine of primer labelling and a switch to nucleotides in order to make assays even more efficient and sensitive.

## Methods

### RPA reaction

*RPA and DNA-Template:* In this study, RPA was performed using the TwistAMP^®^ Liquid Basic Kit (TwistDxTM Limited; TALQBAS01) according to the manufacturer's instructions. Any modifications are noted at the appropriate point. Amplification took place for 60 min during the incorporation rate studies and for 40 min during the microarray experiments. The volume of the reaction mixture was 25.5 µl. For the multiplex microarray experiments, genomic DNA prepared and provided by the Robert Koch Institute was used with resistance genes from different pathogens (*bla*_CTX-M15_ [*E. coli*; 735/14–1], *bla*_NDM_ [*E. coli*; 2/10], *bla*_VIM_ [*P. aeruginosa*; 359/11]; *bla*_KPC_ [*E. coli*; 17/11]) and concentrations up to 1 ng/µl (Table 1). The corresponding concentrations are given in the associated results section. Purified PCR template with a concentration of 1 ng/µl to 1.5 ng/µl was used for the general study of incorporation rates and singleplex RPA.

**Table 1:** List of organisms and primer. Illustration of the organisms used in this study including the target resistance gene and the RPA primer.

Organism	Isolate no	Resistance gene	Primer	Sequence	Fragment [bp]
*E. coli*	2/10	*NDM-1*	NDM-R *	CAAGCTGGTTCGACAACGCATTGGCAT	220
			NDM-F *	CAACGGTTTGATCGTCAGGGATGGCGG	
*P. aeruginosa*	359/11	*VIM-2*	VIM-F *	TGGTCTCATTGTCCGTGATGGTGATGAGTTGCT	191
			VIM-R *	TACGTTGCCACCCCAGCCGCCCGAAGGACATC	
*E. coli*	17/11	*KPC-2*	KPC-F *	CATTCGCTAAACTCGAACAGGACTTTG	809
			KPC-R *	CCAATAGATGATTTTCAGAGCCTTACTG	
*E. coli*	735/14–1	*CTX-M15*	CTX-M15-F *	TCACGCTGTTGTTAGGAAGTGTGCCGCTGTATGC	141
			CTX-M15-R *	CGATAAAGTATTTGCGAATTATCTGCTGTGT	

The isolate number refers to the nomenclature of the Robert Koch Institute. All primer were synthesized and provided by metabion international AG. (*reference: Warmt et al.^33^).

*Cy5 labelling*: For labelling the amplicons with Cy5 fluorescent label, either 5-(3-aminoallyl)-2'-deoxyuridine-5'-triphosphate (Cy5-dUTP) from Jena Bioscience (NU-803-XX-CY5-S) or primer labelled at the 5'-end from metabion international AG were chosen. Again, the respective concentrations are listed at the corresponding position in the results section.

### Amplicon purification, gel analysis and fluorescence spectroscopy

*Purification*: For the detection of the Cy5 molecules, incorporated during RPA, by gel electrophoresis and fluorescence spectroscopy, the RPA products were previously purified using magnetic beads. For this purpose, the Mag-Bind® Total Pure NGS Kit (Omega Bio-Tek; M1378-01) was used according to the manufacturer's instructions. The elution step was performed in 25 µl ddH_2_O.

*Gel electrophoresis*: To view the labelled and unlabelled amplicons, they were separated and analysed according to their size after RPA and appropriate purification on a 2% agarose gel in 1 × TAE buffer (50 × TAE-buffer; PanReac AppliChem; A1691). For detection of all bands and markers, 2 µl peqGreen was added to the gel (VWR; 732–3196). To 15 µl of each DNA-sample, 3 µl of DNA Gel Loading Dye (6 x) (Thermo Fisher Scientific; R0611) was added and electrophoresis was performed at 120 V for 1.5 h. Gene Ruler Low Range DNA Ladder (Thermo Fisher; SM1193) and peqGOLD DNA-ladder mix (VWR; 25–2040) came into use for the size assignment of the gel bands. For the detection of the gels the LS Reloaded Microarray Scanner (Tecan) was used. Here, a custom 3D-printed holder for the gels was used. With this modified method, both the green channel for the recognition of the DNA intercalator (excitation at 532 nm) and the red channel (excitation at 633 nm) for the incorporated Cy5 molecules could be analysed under the same conditions at the same time.

*Fluorescent spectroscopy:* For the determination of the incorporation rates of Cy5 molecules into the RPA products, 20 µl of each previously purified sample was analysed in FLUOstar Omega (BMG Labtech). Excitation at 620 nm and a 680 nm emission filter were used. For the calibration line needed to calculate the incorporation rates, a Cy5-dUTP dilution series in the range of 2.0–20.0 µM was measured under the same conditions.

### Microarray analysis

Microarray analysis was performed using the hybridization protocol published in Warmt et al.^33^.

For this, the DNA-probes were spotted onto 3D-epoxy glass slides (PolyAn GmbH) utilizing a sciFLEXARRAYER SX (Scienion GmbH). After blocking with ethanolamine and subsequent washing steps, hybridization took place at 52 °C for 90 min. Subsequently, several washing steps were performed before the slide was analysed on the GenePix 4300 microarray scanner.

Probe sequences are listed in Table 2.

**Table 2:** List of immobilized probes. Illustration of the specific and unspecific (negative control; NC) probes used for the hybridization and detection of the RPA amplicons via microarray.

Probes	Resistance gene	Sequence
CTX_M15_P200	CTX-M15 **	Spacer-GACTGGGTGTGGCATTGATTA
CTX_M15_P160	*CTX-M15*	Spacer-AGAGTGAAACGCAAAAGCAG
CTX_M15_P175	*CTX-M15*	Spacer-GAATTAGAGCGGCAGTCGG
KPC_S_A	KPC *	Spacer-GATGACAAGAACAGCGAGG
KPC_S_C	KPC-2 *	Spacer-GATGACAAGCACAGCGAGG
KPC_S_G	KPC *	Spacer-GATGACAAGGACAGCGAGG
KPC_S_T	KPC-3 *	Spacer-GATGACAAGTACAGCGAGG
NDM_P_New	NDM **	Spacer-GGACAAGATGGGCGGTAT
NDM_P_New_as	*NDM*	Spacer-ATACCGCCCATCTTGTCC
NDM_P317	*NDM*	Spacer-CCTCAACTGGATCAAGCAG
NDM_P317_as	*NDM*	Spacer-CTGCTTGATCCAGTTGAGGA
OXA_48_P1	NC **	Spacer-CGCTCCGATACGTGTAACTTA
CTX_M14_P223	NC **	Spacer-ACCAGTAAAGTTATGGCGGC
CTX_M14_P252	NC **	Spacer-GCTTAAGCAGAGTGAAACGC
CTX_M14_P260	NC **	Spacer-AGAGTGAAACGCAAAAGCAG
MCR_1_P437	NC **	Spacer-ATTATCCGACTTGGGGCAAG

The spacer consists of an aminohexyl-linker followed by a poly(T)-sequence. All probes were synthesized and provided by metabion international AG. (*reference: Peter et al.^36^; **reference: Warmt et al.^33^).

### Concentration measurements

Concentration measurements were conducted using Nanodrop 2000 (Thermo Fisher Scientific) with 1.5 µl purified RPA products.
